# Supplementary material for: Eltanexor Effectively Reduces Viability of Glioblastoma and Glioblastoma Stem-Like Cells at Nano-Molar Concentrations and Sensitizes to Radiotherapy and Temozolomide
Source: Biomedicines. 2022 Aug 31;10(9):2145. doi: 10.3390/biomedicines10092145 (PMC9496210; doi:10.3390/biomedicines10092145)
Supplement: Supplementary file 1 [file biomedicines-10-02145-s001.zip › biomedicines-1898050-supplementary second proof.pdf]

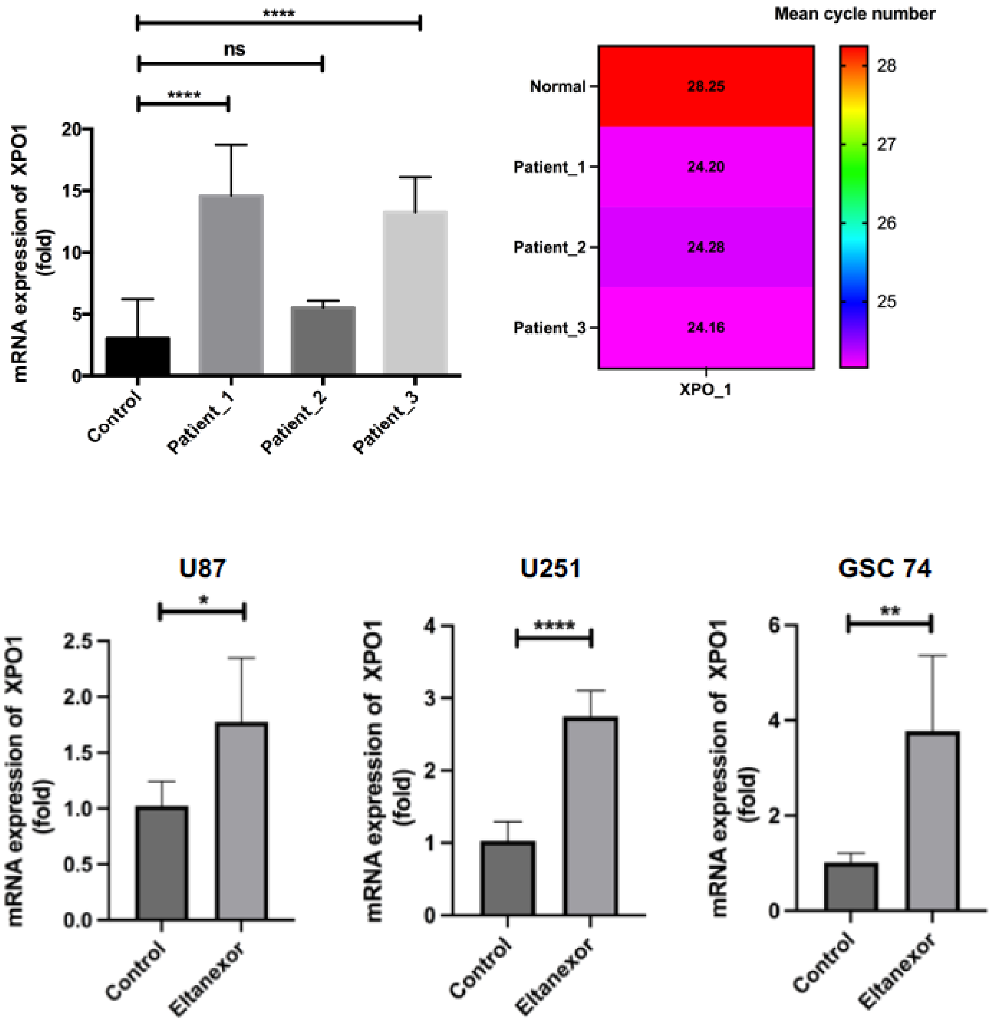

Figure S1: Expression analysis of XPO1 mRNA in normal brain and three representative GBM patients and in GBM cells U87, U251 and GSC 74 after treatment with Eltanexor. * $p < 0.05$, ** $p < 0.01$, and **** $p < 0.0001$.

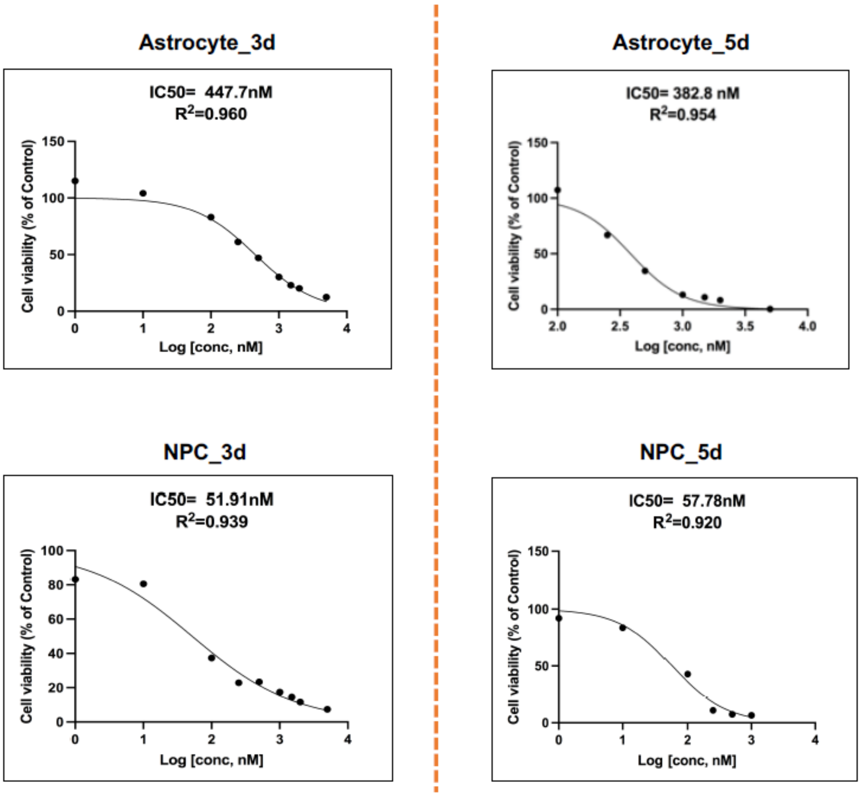

	Astrocyte		NSC	
	3d	5d	3d	5d
IC50 [nM]	447.7	382.8	51.91	57.78

Figure S2. Cell viability curves for Eltanexor tested in primary astrocytes and in neuronal precursor cells (NPC) after 3 and 5 days, respectively. IC_{50} values for Eltanexor were determined and listed in the table below. All data are from three independent experiments performed in triplicates.

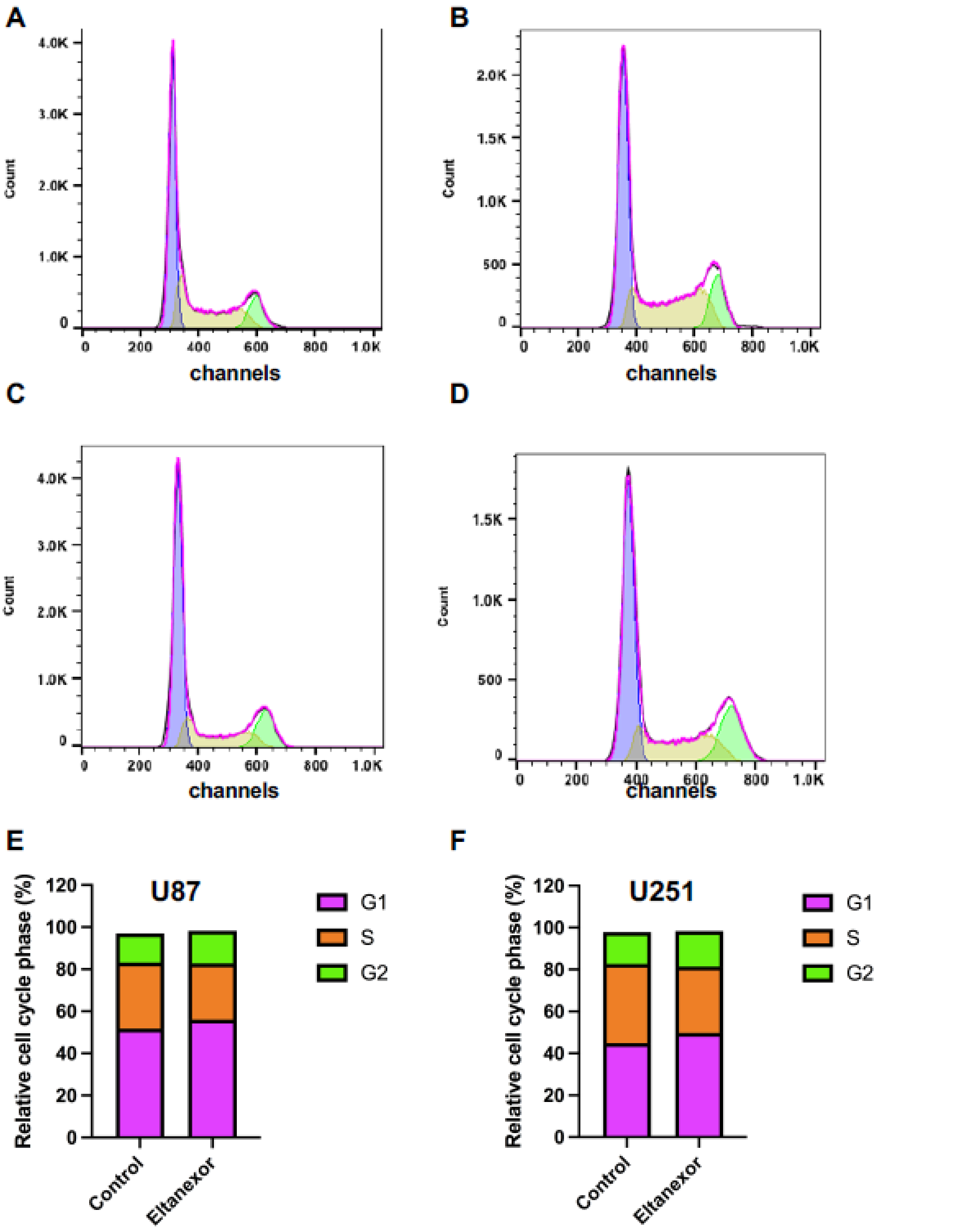

Figure S3: FACS analysis using propidium iodide to determine cell cycle status in U87 (**E**) and U251 (**F**) cells after treatment with Eltanexor (100 nM). Relative parts of cell cycle phases are presented in the histogram (**A–D**).

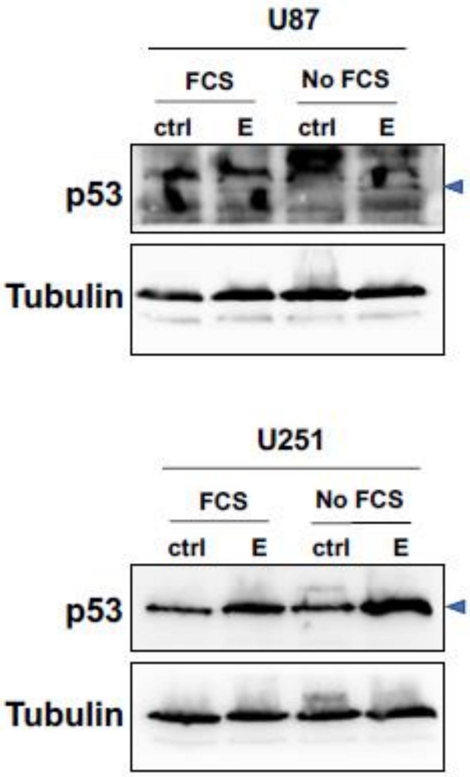

Figure S4: Effect of serum starvation of p53 protein levels in U87 and U251 cells in control and Eltanexor treated cells.

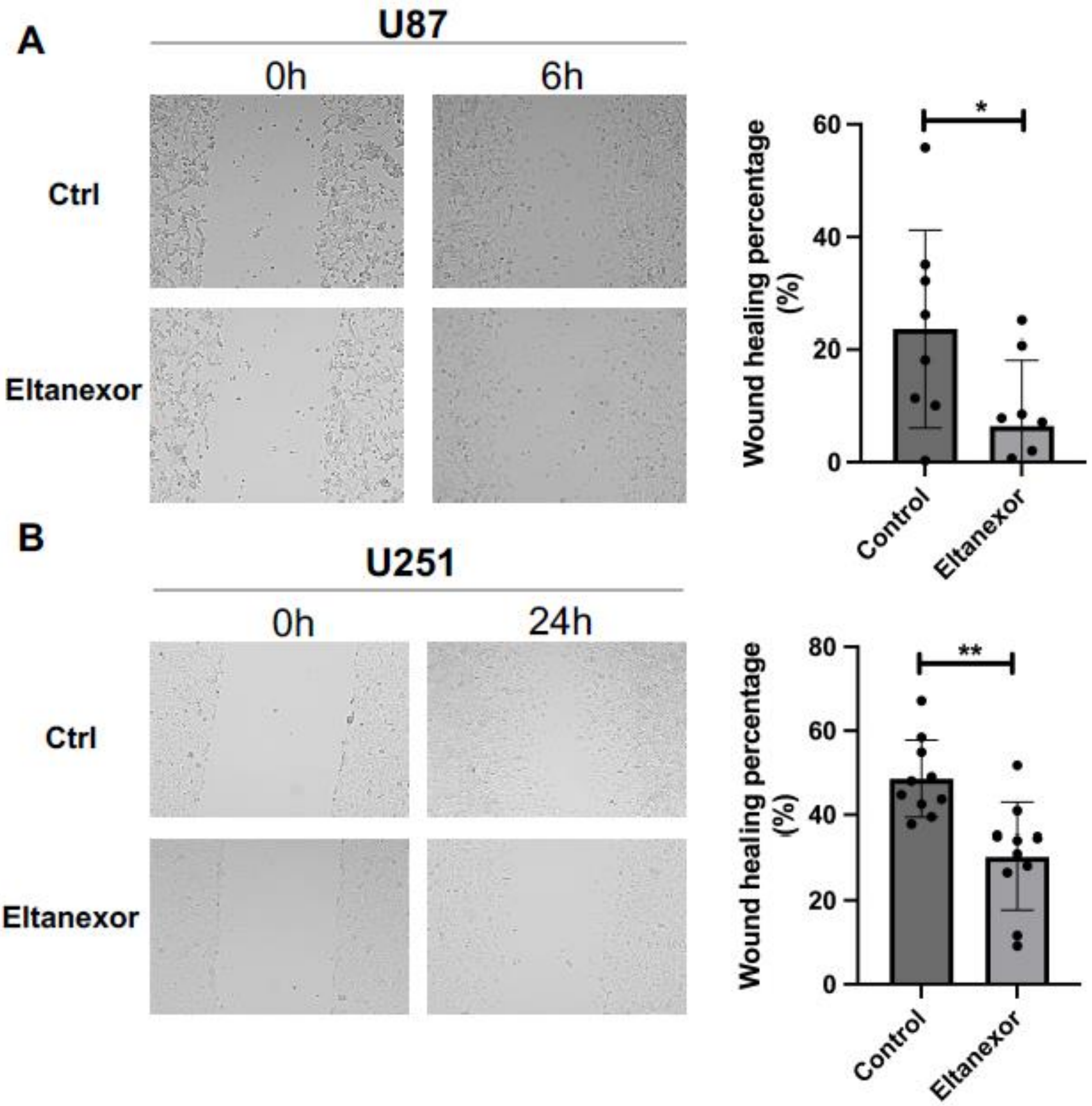

Figure S5: Migration of U87 (**A**) and U251 (**B**) cells after treatment with Eltanexor. Relative cell numbers were determined after 6 h for U87 and after 24 h for U251 cells. * $p < 0.05$, ** $p < 0.01$.
